# *Balanites aegyptiaca* leaf extract-mediated synthesis of silver nanoparticles and their catalytic dye degradation and antifungal efficacy

**DOI:** 10.3389/fbioe.2022.977101

**Published:** 2022-10-04

**Authors:** Anita Dhaka, Shani Raj, Chanda kumari Githala, Suresh Chand Mali, Rohini Trivedi

**Affiliations:** Laboratory of Plant Pathology, Department of Botany, Mohanlal Sukhadia University Udaipur, Udaipur, Rajasthan, India

**Keywords:** silver nanoparticles, green synthesis, *Balanites aegyptiaca*, dye degradation, antifungal activity

## Abstract

This study describes the biosynthesis of silver nanoparticles (AgNPs) using *Balanites aegyptiaca* (*B. aegyptiaca*) leaf extract. The biosynthesized AgNPs were characterized by UV-Vis spectroscopy, Fourier transform infrared spectroscopy (FTIR), dynamic light scattering (DLS), X-ray diffraction (XRD), Raman spectroscopy, transmission electron microscopy (TEM) and scanning electron microscopy with (SEM-EDS). The AgNPs showed an average size of 10–20 nm, spherical shape, and crystalline nature. The application of these synthesized AgNPs to dye degradation showed that the AgNPs removed the two organic pollutants methylene blue (MB, 93.47%) and congo red (CR, (78.57%). *In vitro* investigation of the antifungal activity of the AgNPs against *Fusarium oxysporum*, a phytopathogenic fungus, showed a maximum percent radial growth inhibition of 82.00 ± 1.00% and a spore percent inhibition of 73.66 ± 3.94 for 150 μg/ml of biosynthesized AgNPs.

## Introduction

Nanotechnology involves the design, synthesis, and manipulation of nanoparticles (1–100 nm), although this concept has changed over time (Azarbani and Shiravand, 2020). Interest in nanotechnology has recently sharply increased due to its unique properties in applications in various fields including electronics, the optical industry, and medicine (Das et al., 2013; Gopinath et al., 2020). Their size gives nanoparticles unique physical and chemical properties over their larger counterparts (Verma and Maheshwari, 2019; Raj et al., 2021). Among various synthesized nanoparticles, metal nanoparticles (MNPs) such as silver (Ag), gold (Au), copper (Cu), and zinc (Zn) have attracted interest (Mali et al., 2019; Elshafei et al., 2021). Among MNPs, AgNPs were the first to provide solutions to previously unsolved problems. AgNPs have been used for targeted drug delivery, cancer therapy, biosensing, optical devices, electronics, magnetics, photonics, catalysis, water purification composite fibers, biosensor material, pollutant remediation, wastewater treatment, food packaging material, food storage containers, and cosmetic items (Ahmed et al., 2020; Fiorati et al., 2020; Jildeh and Matouq, 2020; Sridhar et al., 2021). Every metal has two sides; i.e., the advantages and application benefits of NPs. With the advantages come disadvantages, including the methods for NPs synthesis. NPs can be synthesized using physical, chemical, and biological methods. While chemical and physical methods are often used to synthesize AgNPs, they generally require hazardous reactants, high energy consumption, complex purifications, unstable yields, and low conversion efficiency, leading to higher costs and environmental hazards (Barman et al., 2020). The biological approach uses microorganisms such as bacteria (Gopinath et al., 2012; Gopinath et al., 2013; Ajaz et al., 2021), algae (Chugh et al., 2021), fungi (Santos et al., 2021), and plants (Velusamy et al., 2015; Hakimi and Alikhani, 2020; Gudimalla et al., 2021; Okaiyeto et al., 2021). Plants are optimal due to their natural availability, efficiency, low cost, and eco-friendliness (Mustafa et al., 2020), (Dashora et al., 2022). The phytochemicals in plants act as reducing, stabilizing, and capping agents for NP synthesis (Dawoud et al., 2021). Biological methods come to the forefront of synthesis due to their affordable, low-cost, and non-toxic nature. They are also in line with global efforts to eliminate hazardous waste.

The plant source used in the present study was *Balanites aegyptiaca* (Linn.) Del. Zygophyllaceae, commonly known as desert date. This prickly shrub grows into a tree and is widespread throughout Africa and southern Asia. The traditional or ethnobotanical uses of this plant include the treatment of diseases including jaundice, malaria, syphilis, asthma, epilepsy, hemorrhoids, abdominal pain, dysentery, constipation, and fever. Its phytocomponents also include coumarins, sinapic acid, ferulic acid, and organic acids (Al-Thobaiti and Abu Zeid, 2018; Murthy et al., 2021).

Organic dyes are waste products from the textile, plastic, leather, paper, and pharmaceutical industries (Singh et al., 2021). They are highly toxic and carcinogenic, cause allergic reactions and kidney and liver damage, and are mutagenic by damaging the central nervous system (Khodadadi et al., 2017). Industrial effluents discharged into soil and water cause environmental and health problems of great concern (Naseem et al., 2019). While various physical and chemical approaches have been developed and applied in recent years to treat these waste products, they have many disadvantages (Veisi et al., 2018; Sabouri et al., 2021). Biosynthesized NPs have also shown potential for organic dye degradation. AgNPs degrade organic dyes via redox potential approaches and photocatalytic reactions in sunlight (Raj et al., 2020).

Food and crop plants are threatened by many biotic agents, of which phytopathogenic fungi are a major concern. Plant and seed diseases caused by phytopathogenic fungi lead to quantitative and qualitative losses in agriculture (Gopinath and Velusamy, 2013; Kumar et al., 2020; Sabouri et al., 2022a). *Fusarium oxysporum* is widespread in soil-borne fungal communities in all types of soils worldwide. This species is also a natural component of fungal communities in the rhizosphere of plants. All strains of *F. oxysporum* are saprophytic, suggesting that they can grow and thrive on organic materials in the soil and rhizosphere of many plant species for long periods of time. In addition, some strains of *F. oxysporum* are harmful to a wide range of plant species; When they reach the vascular system, they cause root rot or tracheomycosis (Fravel et al., 2003). To avoid losses, the use of synthetic fungicides has increased. Their negative effects include the impairment of human and environmental health. Therefore, biological approaches are urgently needed for sustainable agricultural growth (Dutta et al., 2022). NPs have also shown promise in crop protection (Mali et al., 2020; Sabouri et al., 2022b; Salem et al., 2022). However, despite reports and studies, additional evaluation is needed.

In this study, we report the synthesis of AgNPs via biological methods using the leaf extract of *B. aegyptiaca*. The synthesized AgNPs were characterized using different analytical techniques. Finally, the synthesized AgNPs were also used as a catalyst for the degradation of MB and CR and as an antifungal agent against *F. oxysporum* in *in vitro* studies.

## Materials and methods

### Materials

Chemicals including AgNO_3_ (Sigma-Aldrich, St. Louis, United States), NaBH_4_ (Sigma-Aldrich), and MB and CR dyes (HiMedia, India) were purchased from Pvt. Ltd. New Delhi (India). Potato dextrose agar (PDA) was purchased from HiMedia. *F. oxysporum* (ITCC #4998) was purchased from IARI, New Delhi. Fresh and healthy *B. aegyptiaca* (Linn.) leaves were collected from Udaipur, Rajasthan (India). The collected plant material was authenticated by the Herbarium, Botany Department, University of Rajasthan, Jaipur, India (#RUBL211432). Autoclaved deionized water (DIW) was used throughout the experimental process.

### Preparation of the plant extract

Fresh and healthy leaves were harvested, washed fully with tap water and then deionized water to remove all dust and visible particles, cut into small pieces, and dried in the shade at room temperature for 2 weeks. The dried leaves were then ground into a fine powder with an electric mixer. Five grams of powder sample was then mixed in 200 ml of deionized water and the mixture was boiled at 70°C in a serological water bath for 30 min. Thereafter, the extract was filtered through Whatman No. 1 filter paper (HiMedia) to remove particulate matter and obtain a clear solution. The filtrate solution was kept at a low temperature (4°C) for further use.

### Silver nanoparticle synthesis

For AgNP synthesis, 10 ml of filtrate was mixed with 90 ml of 1 mM AgNO_3_ solution. To reduce the photo-oxidation of AgNO_3_, the synthesis process was performed under low light. The pH of the reaction mixture was adjusted to 9 using 0.1N NaOH and 0.1N HCl. The formation of AgNPs was confirmed by the color change of the reaction solution and spectrophotometric analysis. The prepared solution was centrifuged at 15,000 rpm for 20 min at 4°C. The transparent solution was then discarded and the AgNP pellets were collected. The pellets were washed three times with deionized water to remove impurities and then oven dried at 40–50°C.

### Characterization of the silver nanoparticles

UV-Vis spectroscopy was used for the preliminary identification of AgNP formation. A UV-Vis spectrophotometer (Systronics 117 UVvisible Spectrophotometer) was used to record the extinction spectra of the AgNPs at 300–650 nm at a 1-nm resolution using a quartz cuvette cell with 1 cm path length. The reaction of AgNO_3_ with plant extract was optimized spectrophotometrically by adjusting various parameters, including AgNO_3_ concentration, pH, temperature, and time.

The chemical composition of the synthesized AgNP was determined using an FTIR spectrometer (Bruker, United States) at room temperature. FTIR spectroscopy was performed to identify the biomolecules present in the leaf extract responsible for the reduction of AgNO_3_ to AgNPs. The permeability was recorded at 500–4000 cm^−1^.

The crystal structure and particle size of the AgNPs were determined by XRD using an X-ray diffractometer (Ultima IV, Rigaku, Japan) at room temperature. A dried powder of the synthesized AgNPs was analyzed by XRD using CuK radiation (1.54 nm) with a scan angle of 2θ, ranging from 20° to 90°.

DLS was used to determine the particle size distribution, polydispersity index (PDI), and zeta potential of the synthesized AgNPs. DLS was performed using a Malvern Zetasizer (Malvern Instrument Inc., London, UK) at 25°C and a scattering angle of 90°. The size range was recorded from 0.1 to 10,000 nm.

The Raman spectrum of the synthesized NPs was recorded at room temperature with a 532 nm laser. The AgNPs were scattered over the slide. The solvent was then evaporated to form a thin film of AgNPs for Raman analysis. The thin film was exposed to a laser beam with a spectral range of 0.3000 cm^−1^ for 1000 s. The scattered light was collected and detected on a Iso Plane SCT-320, PIXIS 100 Princeton instrument MNIT Materials Research Center [MRC], Jaipur (Raj.).

Electron microscopy was performed for the morphological examination of the synthesized NPs on a TEM instrument (TEM-Tecnai G220, United States). SEM (JEOL SM-7600F Japan) was performed coupled with an X-ray energy dispersive spectrometer (Oxford EDS system). The surface morphology of the biosynthesized AgNPs was studied by SEM. EDS was used to determine the elemental composition of the AgNPs. The EDS spectra were processed using INCA Microanalysis Suite. High-resolution (HR)-TEM was also used to characterize the AgNPs. HR-TEM was performed at 200 kV to determine the AgNP shape, size, and morphology, as well as the elemental composition and selected area electron diffraction (SAED) pattern. A drop of colloidal AgNPs was placed on a carbon-coated Cu grid, which was then dried at room temperature imaging on a microscope.

### Catalytic degradation of dyes

In the presence of NaBH_4_, the catalytic activity of the synthesized AgNPs was assessed by degrading the hazardous dyes MB and CR. The synthesized AgNPs were sonicated for 15 min by Ultra-Probe sonication to prepare an aqueous colloidal suspension. The catalytic study using the synthesized AgNPs for MB and CR degradation was performed as described in Supplementary Table S1. The reaction mixture was placed in a quartz cuvette cell (1 cm path length) and the kinetics were monitored using a UV-Vis spectrophotometer at 664 and 490 nm. A change in the absorption peak was observed due to the change in the concentrations of MB and CR over time at 200–800 nm. The absorption spectrum was recorded at 1-min intervals.

The reduction process was performed in a 4 ml quartz cuvette and the absorption spectra were recorded to monitor the time-dependent reduction time. A blank without AgNP was used as the reference. The pseudo-first-order kinetics were calculated as follows:

$$\ln\left(A_{t}/A_{0}\right)=-kt$$ (1)

Dye degradation was expressed using the following equation:

$$Degradation\;\%=\frac{A_{0}-A_{t}}{A_{0}}\times 100$$ (2)

where A_0_ = initial absorbance of the dye, A_t_ = absorbance of the dye at time t, and *k* = rate constant. The entire procedure was performed at ambient temperature.

### Antifungal activity of silver nanoparticles

The antifungal activity of the biosynthesized AgNPs against *F. oxysporum* was evaluated using the poison food technique. In this study, six treatments (one control with water; plant extract; 150 μg/ml AgNO_3_; and 50, 100 and 150 μg/ml (w/v) AgNPs) and 100 μg/ml Bavistin (a commercial fungicides used as positive control), were used to assess the antifungal activity. Four replicates per treatment were used and all plates were incubated at 28 ± 2°C for 7 days. Colony diameters were measured daily and the data were used to calculate the potency. The percent inhibition rate of mycelia was calculated using the formula given by Vincent (Vincent, 1947), as follows:

$$Inhibition\;rate\;\%=\frac{mycelial\;growth\;in\;control-mycelial\;growth\;in\;treatment\;}{mycelial\;growth\;in\;control}\times 100$$ (3)

### Spore germination

The antifungal effects of 50, 100, and 150 μg/ml of synthesized AgNP on spore germination were examined. A spore suspension of 4.0×10^4^ of *F. oxysporum* was prepared aseptically from a 7-day-old culture maintained on PDA at 28°C. The concentration of spores/ml was determined with a hemocytometer. A spore suspension of 50 µl + 50 µl of synthesized NPs in an aqueous solution at the concentrations indicated above was placed on a well glass slide. Three replicates were performed for each concentration. All treatments were maintained at 28°C for 10 h. The percent inhibition rate was calculated by counting the number of spores that germinated compared to the control.

$$Inhibition\;rate\;\%=\frac{Germination\;in\;control\;–\;germination\;in\;treatment}{germination\;in\;control}\times 100$$ (4)

### Statistical analysis

Statistical analysis was performed using analysis of variance (ANOVA), followed by Tukey HSD tests (*p* = 0.05) using IBM SPSS Statistics for Windows, version 26.0. Microsoft Office, OriginPro 2020, and Corel Draw were used to calculate and produce the graphs and figures.

## Results and discussion

### UV-visible spectroscopy analysis

**FIGURE 1:**
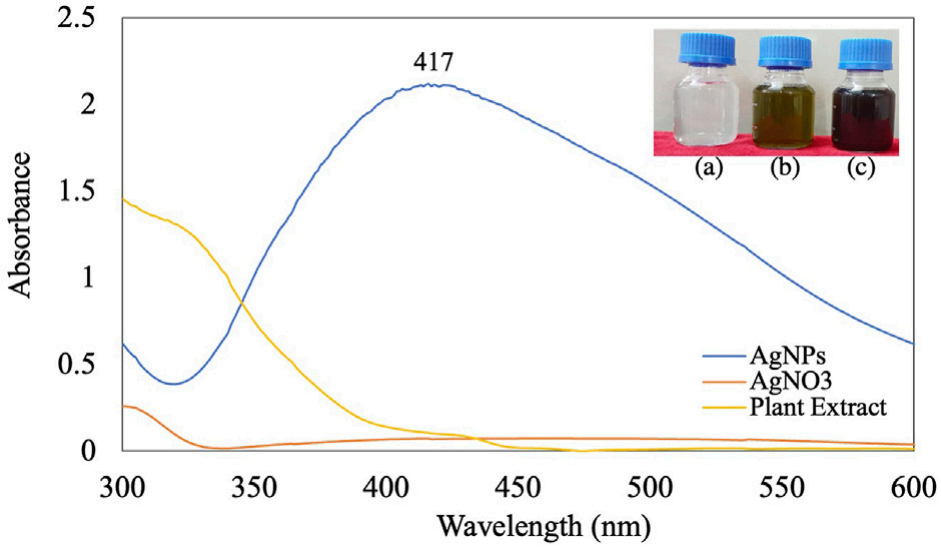
UV-visible spectra of synthesized AgNPs [inlet **(A)** AgNO_3_; **(B)** plant extract; **(C)** AgNPs].

The addition of *B. aegyptiaca* extract to the AgNO_3_ solution resulted in a color change of the solution from light green to dark brown within 5 min at pH 9 (Figure 1). The dark brown color could have occurred due to the free electrons of AgNPs collectively oscillating in resonance with the frequency of light wave interactions, causing a surface plasmon resonance (SPR) band in the visible and infrared spectrums (Okaiyeto et al., 2021). UV spectrophotometric analysis of the synthesized AgNPs showed -max values of 400–430 nm. Previous studies reported absorption bands of spherical NPs at 400–420 nm (Gudimalla et al., 2021). The leaf extract in the present study reduced AgNO_3_ to AgNPs, as confirmed by the absorption peak at 417 nm (Al-Zaban et al., 2013; Sabouri et al., 2022c).

### Optimization of silver nanoparticle synthesis

**FIGURE 2:**
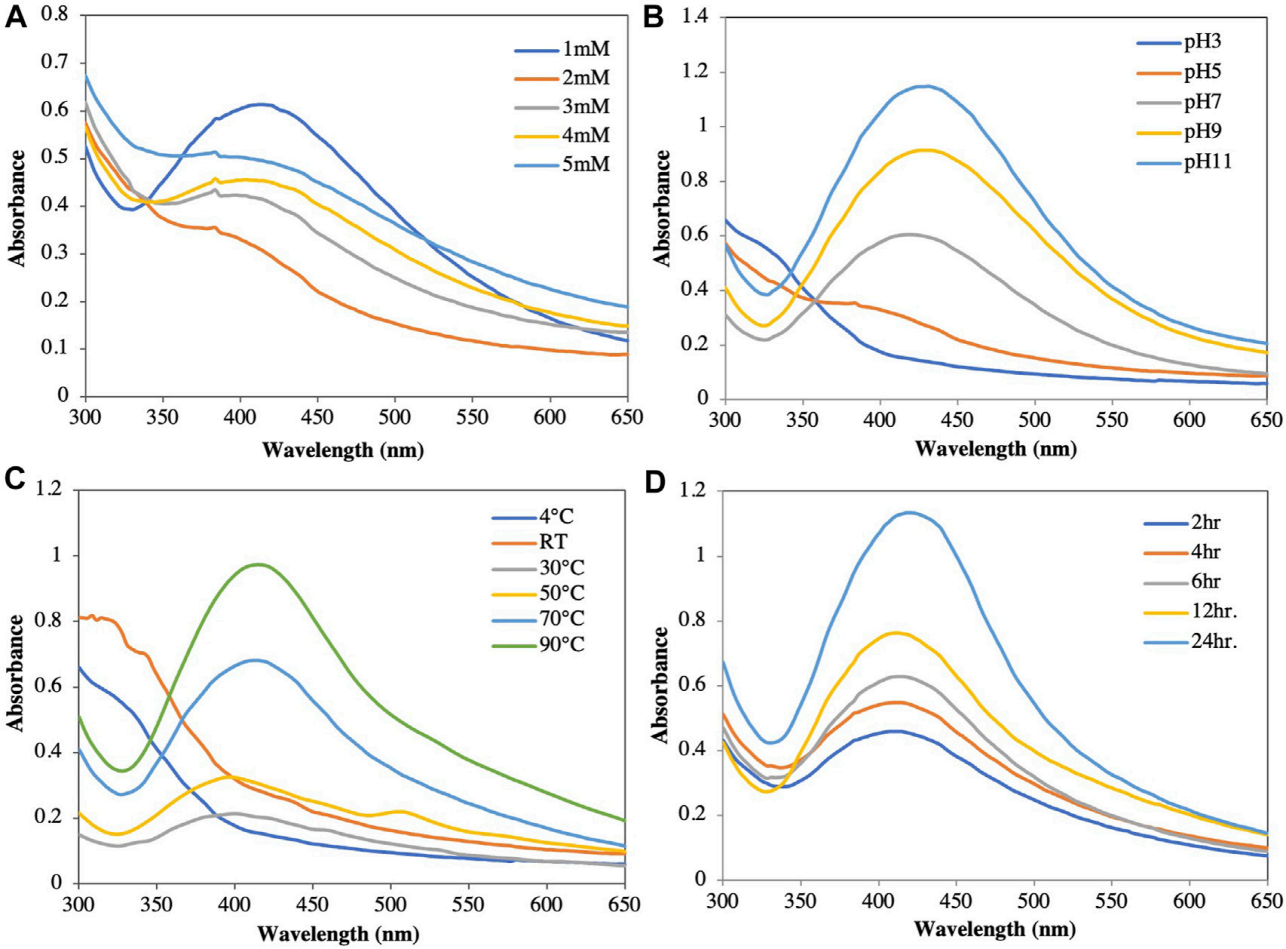
Optimization of synthesis of silver nanoparticles at different parameters. **(A)** AgNO_3_ concentration. **(B)** pH. **(C)** Temperature. **(D)** Time duration.

*AgNO*_*3*_ *concentration:* In this study, 1, 2, 3, 4, and 5 mM AgNO_3_ were used to optimize the concentration at pH 9. The estimated optimal concentration of AgNO_3_ was 1 mM based on UV-Vis scanning. At higher concentrations, the reaction became saturated due to large particles after coagulation (Figure 2A). The optimal silver ion concentration for the preparation of AgNPs using *Annona squamosa* peel extract was also 1 mM (Kumar et al., 2012).

*pH:* The color of the solution and the intensity of the absorbance increased as the pH increased from acidic to basic; however, no characteristic peak was observed at acidic pH values (pH 3 and pH 5). At a neutral pH of 7, the reduction started but at a very slow rate, Increasing the pH towards alkaline showed an immediate reduction after the addition of the plant extract to the AgNO_3_ solution, as well as a color change of the solution from yellow to reddish brown. While the color formation was rapid at pH 11, agglomeration was observed immediately after the addition of silver nitrate, which remained stable for no more than 1 week. At pH 9, the nanoparticle stability was higher than those at pH 3, 5, 7, and 11 (Figure 2B) (Mortazavi-Derazkola et al., 2021). This finding was comparable to that reported by Konwarh et al. (2011), who reported a slower AgNP production and aggregation at acidic pH values. In contrast, Edison and Sethuraman (2012) reported that Ag^+^ precipitated as AgOH at basic pH values. Therefore, pH values play a crucial role in the reduction and stability in nanoparticle synthesis (Wei et al., 2021; Kang et al., 2022).

*Temperature:* When NP solutions were stored at different temperatures (4°C, 30°C, 50°C, 70°C, 90°C, and room temperature [RT]) and evaluated spectrophotometrically, the absorbance and color intensity of the solution increased with increasing temperature (Figure 2C). While the maximum absorbance was observed at 90°C, the NPs agglomerated and settled. This agglomeration could be attributed to the aggregation of smaller particles over long periods of time and at high temperatures (Pourmortazavi et al., 2015; Arya et al., 2018). At low temperatures, the reduction rate was slow and did not show an optimal peak. In our study, the optimal temperature was 70°C, which showed a sharp spectral peak. Moreover, the particles were suspended in solution with high stability for a long time.

*Time:* The amount of NPs produced was determined by the length of time that the AgNO_3_ interacted with the plant extract. Increasing the reaction time resulted in increased color intensity with the incubation duration and a gradual increase in absorption spectra with a surface plasmon resonance of 417 nm after 24 h of incubation (Figure 2D) (Manosalva et al., 2019; Elshafei et al., 2021). After 24 h incubation in a dark room at 30°C, assessment of the stability of the AgNPs biosynthesized in this study showed no further changes in the peak.

### Fourier transform infrared spectroscopy analysis and Raman spectroscopy

**FIGURE 3:**
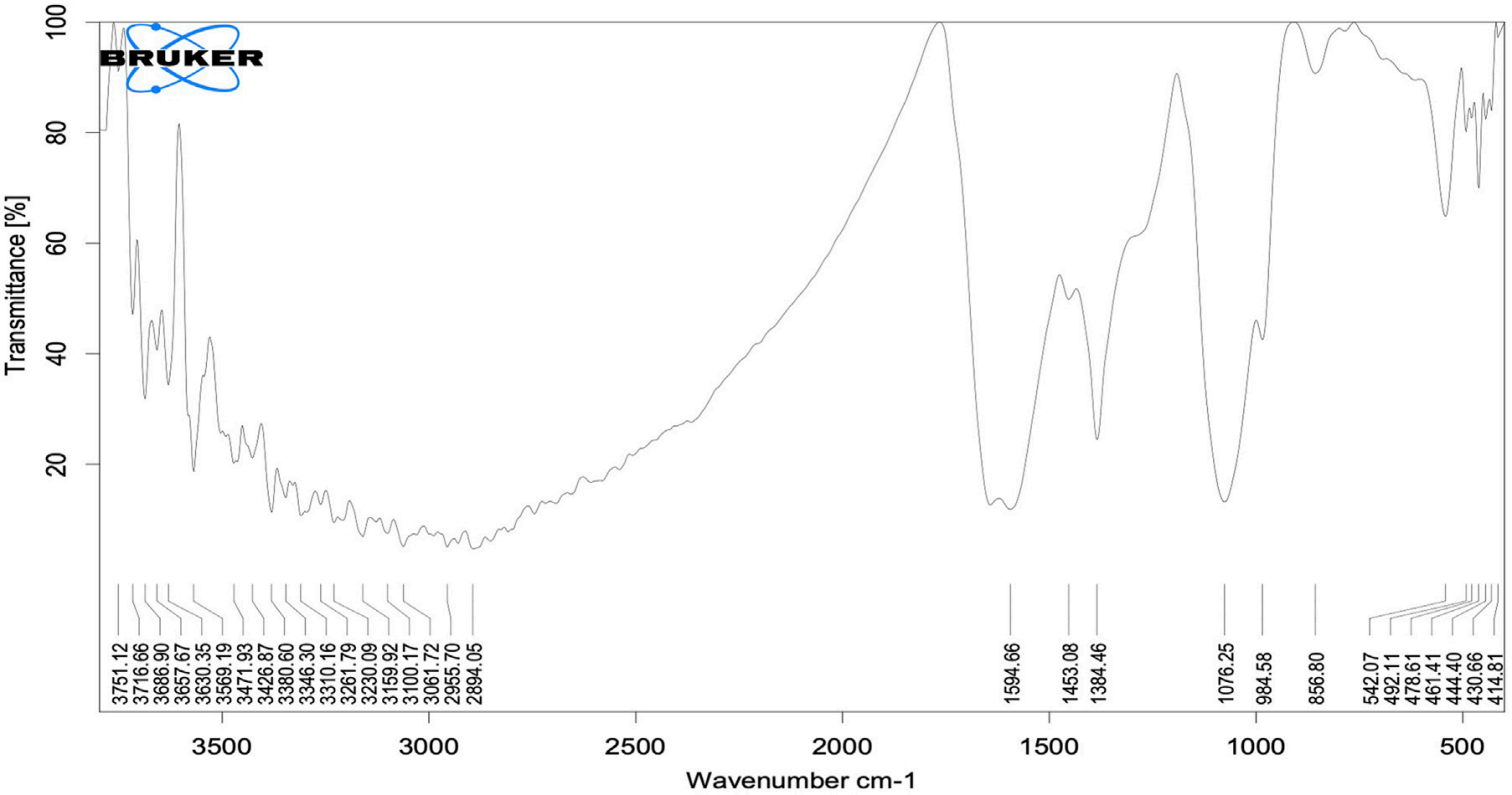
FTIR spectra of synthesized AgNPs.

The biomolecules in the leaf extract that caused Ag^+^ reduction and acted as capping agents for efficient stabilization were identified by FTIR spectrum analysis. Absorption peaks at 3686, 3360, 3061, 1594, 1384, 1076, and 984 cm^−1^ were visible in the FTIR spectra of the biosynthesized AgNPs (Figure 3). In the spectrum, the absorption peak at 3686 cm^−1^ resulted from O-H stretching due to the presence of alcohol and phenol groups. The peak at approximately 3360 cm^−1^ was related to the stretching of N-H bonds resulting from aliphatic primary amines (Jain and Mehata, 2017). The 3061 cm^−1^ C-H bond stretch occurred due to plant metabolites, while the 1594 cm^−1^ peak of N-O stretch derived from a nitro compound. The peak at 1384 cm^−1^ corresponded to the C-H stretching frequencies of the alkene group. The band at 1076 cm^−1^ represented a very strong S=O stretch, consistent with sulfoxides, while the band at 984 cm^−1^ represented a C=C stretch, strongly indicative of the presence of alkene groups (Nallal et al., 2021). Thus, FTIR amine (N-H), hydroxyl (OH), and sulfinyl (S=O) groups in the leaf extract bioactive compound led to a reduction of Ag^+^ ions to Ag^0^.

**FIGURE 4:**
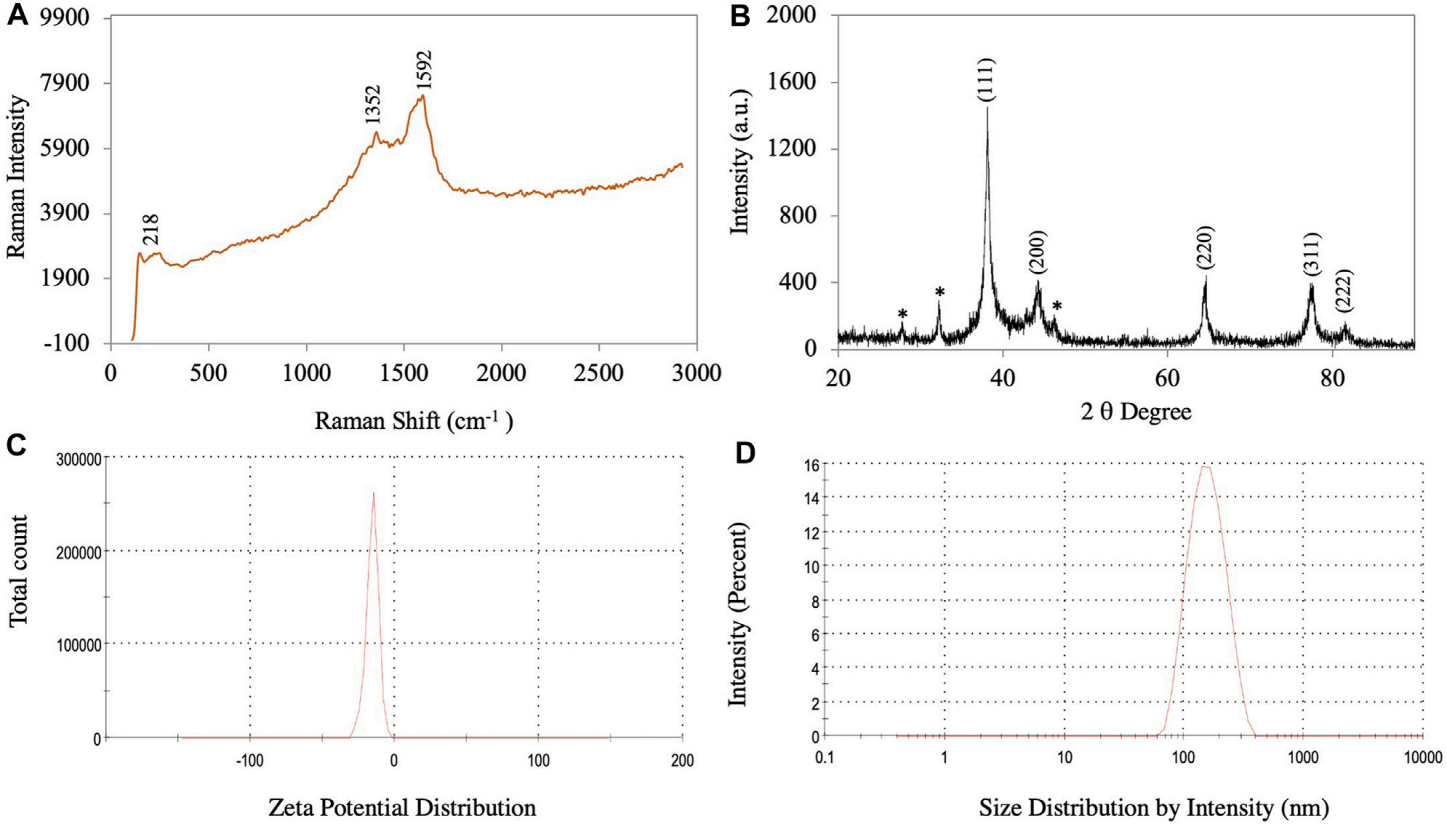
**(A)** Raman spectroscopy of synthesized AgNPs. **(B)** XRD pattern of synthesized AgNPs. **(C)** Zeta potential. **(D)** Size distribution of synthesized AgNPs.

Only three peaks were visible in Raman spectra of the synthesized AgNPs: 218, 1352 and 1592 cm^−1^ (Figure 4A). These peaks showed the interaction of the leaf extract with AgNO_3_. The peaks at 1352 and 1592 cm^−1^ indicated the presence of AgNPs (Albeladi et al., 2020). The Raman spectra of AgNPs showed a group of vibrational peaks at 1592, 1352, and 218 cm^−1^. The main positional peak was observed at 1592 cm^−1^ and corresponded to the N-H stretching vibrations (Logaranjan et al., 2016). The peaks at 1352 cm^−1^ belonged to the C-H stretches, while the faint peak at 218 cm^−1^ was related to C-C stretches (Neethu et al., 2018).

### X-ray diffraction analysis

The crystalline nature of the synthesized AgNPs is confirmed based on the XRD pattern. Figure 4B shows an X-ray diffractogram of the synthesized AgNPs. The XRD pattern showed a Bragg plane of reflection in the 2θ range of 20°–90°. The XRD diffraction peaks in degree 2 appeared at 38.12, 44.24, 64.38, 77.30, and 81.42, which were attributed to planes (111), (200), (220), (311), and (222) sets of lattice planes consistent with the face center cubic (FCC) crystal structure of the AgNPs (Bahrami-Teimoori et al., 2017). The highest intensity peak for FCC materials was generally a (111) reflection in the biosynthesized NPs. Smaller crystalline NPs resulted in broad peaks. The Braggs diffraction peaks agreed well with the database of the Joint Committee on Powder Diffraction Standard of Ag (JCPDS Card No. 04–0783) (Raj et al., 2018). Similar results regarding the Braggs reflection of AgNPs were reported previously (Nouri et al., 2020; Dawoud et al., 2021; Elshafei et al., 2021). The crystallite size of the AgNPs was calculated using the Scherrer equation:

D=kλ/β cos⁡θ (5)

where D = average crystal size (Å), *k* = constant equal to 0.9, λ = X-ray source, Β = angle line full width at half maximum (FWHM), and θ = Bragg angle. The results showed a crystallite size of AgNPs of approximately 17.12 nm.

### Dynamic light scattering analysis

The particle size distribution and surface zeta potential of synthesized AgNPs in an aqueous colloidal solution were determined using the DLS technique. In the present study, the negative zeta potential and zeta deviation were −15.3 mV and 5.22 mV, respectively (Figure 4C). For AgNPs, the ±30 mV zeta potential range is the most stable (Padalia et al., 2015). The high negative value indicated that the synthesized AgNP did not agglomerate (Albeladi et al., 2020). The mean size of the synthesized AgNPs was 149 nm and the polydispersity index (PDI) value was 0.176 (Supplementary Figure S1). The particle size distribution curve of the synthesized AgNPs is shown in Figure 4D. The size distribution results from the DLS analysis show a larger size of the AgNPs compared to those determined by TEM, SEM, and XRD because the biomolecule and water layers covering the surface of the NPs were also included (Ghojavand et al., 2020).

### TEM and SEM-EDS analysis

**FIGURE 5:**
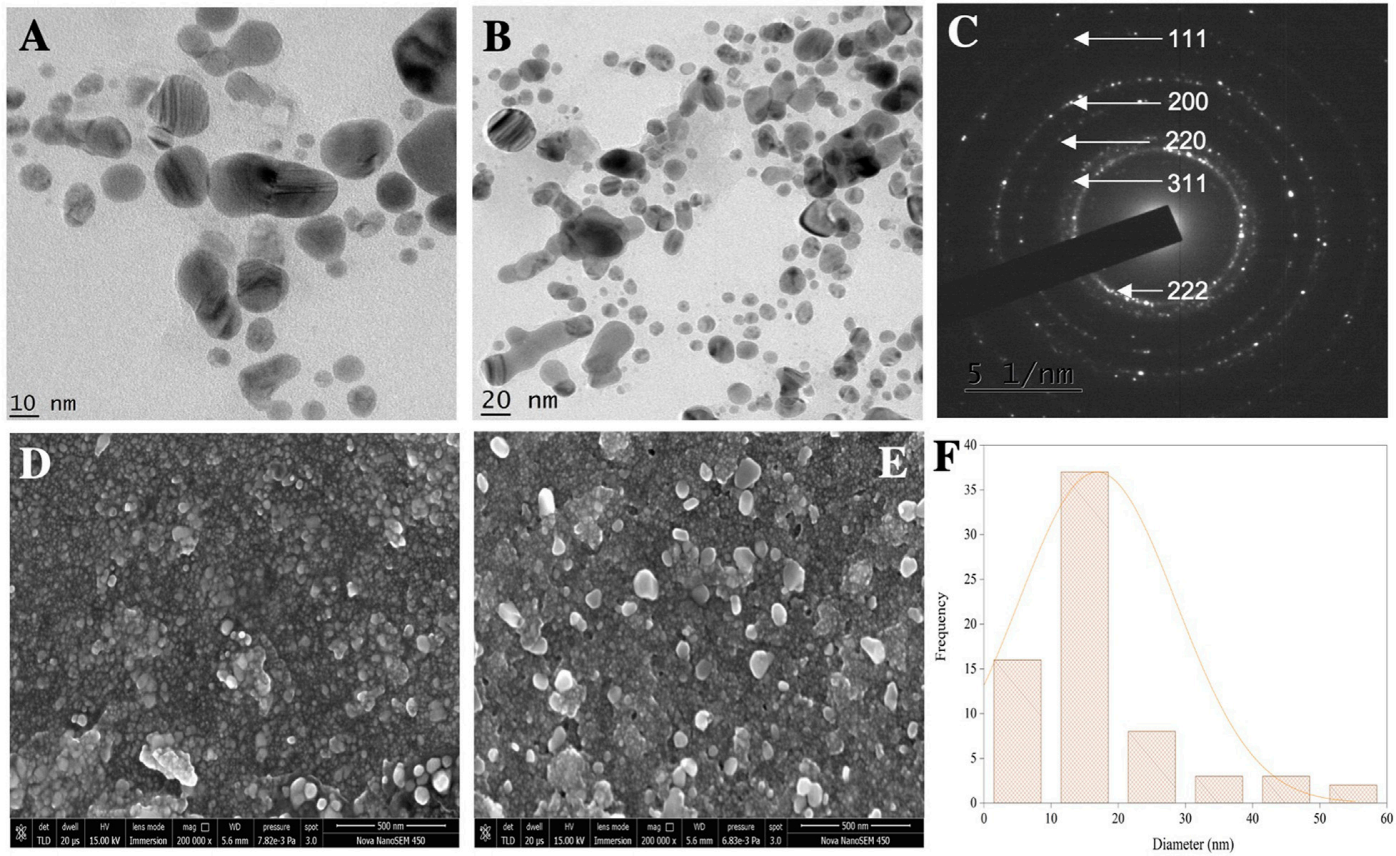
**(A,B)** TEM micrographs of AgNPs. **(C)** SAED pattern. **(D,E)** SEM micrographs. **(F)** Histogram showing the average particle size distribution of synthesized AgNPs.

HR-TEM and FE-SEM techniques were used to confirm the structure, shape, and crystallinity of the biosynthesized AgNPs. To identify individual particles, HR-TEM images were recorded at different magnifications (Figures 5A,B). The biosynthesized AgNPs were spherical and irregular in shape, with small clusters of particles due to agglomeration during sample preparation (Al-Otibi et al., 2021). The distance between the lattice fringes of AgNPs was 0.25 nm (Supplementary Figure S2) (Nguyen et al., 2020). The selected area electron diffraction (SAED) pattern of the biosynthesized AgNPs is shown in Figure 5C. The bright spots corresponding to Braggs reflection planes of (111), (200), (220), (311), and (222) in these patterns were correlated with powder XRD and FCC properties (Figure 5C) (Nallal et al., 2021). The histogram showed many particles ranging in size from 11 to 20 nm (Figure 5F).

**FIGURE 6:**
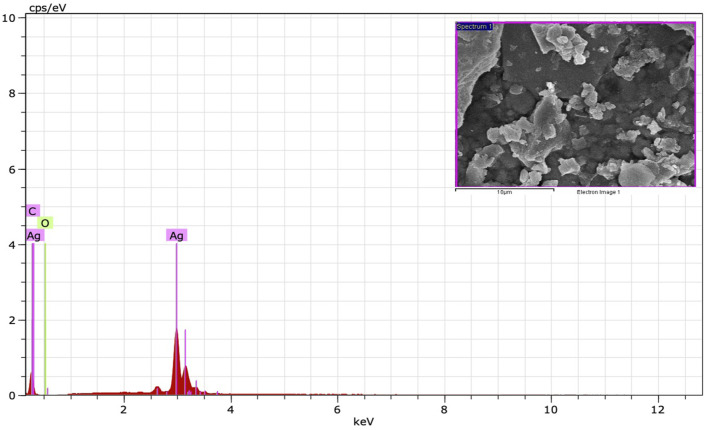
EDS spectrum of synthesized AgNPs.

The FE-SEM analysis revealed spherical particles with a rough surface likely due to an organic layer acting as a capping agent. HR-TEM and FE-SEM assessments were used to determine the particle size (average 10–20 nm) (Figures 5D,E). The presence of metallic Ag was confirmed by EDS to identify the composition of the sample (Figure 6). Metallic Ag generally shows a typical strong peak at 3 kV due to surface plasmon resonance (Das and Velusamy, 2013). The EDS spectrum highlighted the presence of Ag (88.68%) and lesser amounts of other elements such as O (0.77%) and C (10.55%) weight % and Ag (47.02%), O (2.75%), and C (50.23%) atomic %. As shown in Figure 6, the other elements acted as organic capping agents bound to the surface of the AgNPs, comparable to previously reported results (Albeladi et al., 2020; Al-Otibi et al., 2021).

### Catalytic activity of silver nanoparticles

**FIGURE 7:**
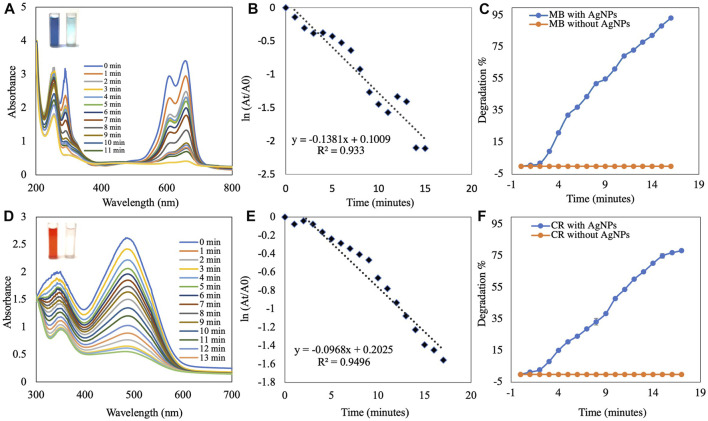
UV-visible absorption spectra analysis of **(A)** catalytic degradation of MB by NaBH_4_ in the presence of AgNPs and **(B)** pseudo-first-order plot of ln(A_t_/A_0_) vs. time of MB. **(C)** Percent degradation of MB over time by AgNPs. **(D)** Catalytic degradation of CR by NaBH_4_ in the presence of AgNPs. **(E)** Pseudo-first order plot of ln(A_t_/A_0_) vs. time of CR. **(F)** Percent degradation of CR over time by AgNPs.

The AgNP-mediated catalytic reduction of MB and CR was investigated in the presence of NaBH_4_ as the reducing agent. MB is a basic dye used in biology, chemistry, and medicine. It is a heterocyclic aromatic chemical compound. In water, the UV-visible band of the MB monomer occurs at 665 nm, which corresponds to the n→π* transition of MB (Naseem et al., 2020;Das and Velusamy, 2014). MB and NaBH_4_ exchange electrons during reduction, with NaBH_4_ acting as a donor and MB as an acceptor. The activation energy plays an important role in the chemical reaction. The addition of synthesized AgNPs to the reaction mixture resulted in the formation of a potential intermediate between the MB dye and the BH_4_^−^ ions, which enhanced the dye-ion interaction. Since the reduction reaction of the AgNPs has a lower activation energy, electron transfer between them is more efficient (Singh et al., 2019). Experiments without AgNPs (control) showed very little reduction in MB over time. The results showed that NaBH_4_ does not reduce MB efficiently and the reaction speed is extremely slow. When biosynthesized AgNPs were added to the mixture, the rate of reduction increased. When MB is reduced to leucomethylene blue (LMB), the blue color of the oxidized form becomes colorless (Naseem et al., 2020; Nouri and Haddioui, 2021). The reduction of MB to LMB (colorless MB) by synthesized AgNPs with NaBH_4_ was evaluated spectrophotometrically at 665 nm, as was the decrease in absorbance (Figure 7A). The reduction reaction was complete within 15 min. The importance of synthesized AgNPs as a catalyst in the reduction process was also confirmed in a pseudo-first-order kinetic diagram (Figure 7B), with a rate constant of *k* = 0.138 min^−1^ (Kumavat and Mishra, 2021). The synthetic dye CR is a toxic and carcinogenic metabolite found in the textile, paper, and rubber industries that causes bladder cancer in humans (Albeladi et al., 2020). The complicated structure and the presence of a diazo group results in physicochemical, thermal, and optical stability, which makes CR difficult to biodegrade. A UV-Vis spectrophotometer was used to monitor the CR degradation reaction. The SPR band associated with the azo group was seen at 498 nm (π→π*) and 338 nm (n→π*) in an aqueous CR solution (Ismail et al., 2018). The azo (N=N) bonds in the dye molecule are broken down during the CR reduction process, resulting in various aromatic amine products. The color of the CR dye changed from bright radish brown to colorless. This color change was monitored by a gradual decrease in the peak intensity of the CR dye solution at a -max of 498 nm (Figure 7D). The reduction reaction was complete within 17 min. The importance of synthesized AgNPs as a catalyst in the reduction process was also confirmed by a pseudo-first-order kinetic diagram (Albeladi et al., 2020; Varadavenkatesan et al., 2020) with a rate constant of *k* = 0.096 min^−1^ (Figure 7E). The percent of dye degradation, as calculated using Eq. 2, showed a degradation >75%. Figures 7C,F show percent degradations of MB and CR of 93.43% and 78.94%, respectively. The synthesized AgNPs offer more catalytic sites and lower activation energy due to their high volume-to-surface ratios. Similar results were also previously reported in studies on the dye degradation activity of synthesized AgNPs (Supplementary Table S2).

The results of the Langmuir–Hinshelwood model suggested that the catalytic degradation of organic dyes occurred due to a surface reaction between the reactant and AgNPs (Supplementary Figure S3) (Raj et al., 2020). In this scenario, NaBH_4_ serves as both an electron donor and a hydrogen supplier. AgNPs act as an intermediate to transfer electrons between the BH_4_^−^ ion and the dye due to their high negative potential (Edison and Sethuraman, 2012; Edison et al., 2016). Upon addition of NaBH_4_ to a solution containing dye and AgNPs, the BH_4_^−^ ions of NaBH_4_ and dye molecules adsorb onto the surface of the AgNPs, resulting in instantaneous electron and hydrogen transport. Diffusion between the adsorbed molecules causes desorption of the colorless degraded by-product, which might yield additional catalytic sites for MB degradation due to the broad surface area of AgNPs.

### Antifungal assay of silver nanoparticles

**FIGURE 8:**
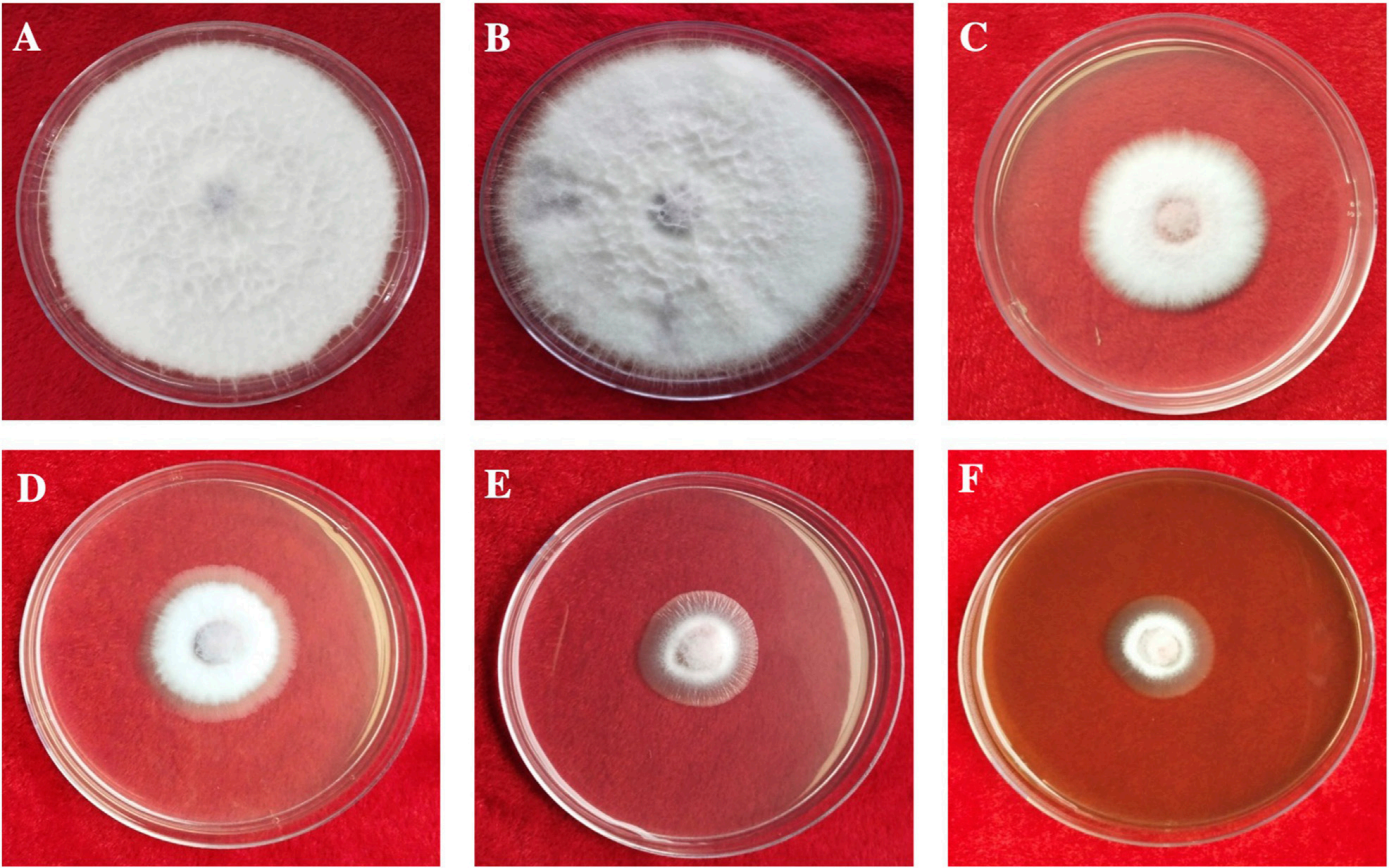
*In vitro* antifungal activity of synthesized AgNPs. **(A)** Control. **(B)** Plant extract. **(C)** AgNO_3_. **(D–F)** 50, 100, and 150 µg/ml of AgNPs.

The antifungal activity of the synthesized AgNPs was determined by measuring radial mycelial growth against *F. oxysporum*. The growth was inhibited by 60.67 ± 2.51%, 72.67 ± 1.52%, and 82.00 ± 1.00% at 50, 100 and 150 μg/ml of aqueous AgNP. The inhibition of mycelial growth at 50, 100, and 150 μg/ml is shown in Table 1. The maximum inhibition of mycelial growth was observed at 150 μg/ml AgNPs. The experiment proved that NP concentrations affect the inhibition of mycelial growth. The commercially available fungicide Bavistin (100 μg/ml) was used as a positive control, which showed 100% inhibition of fungal mycelial growth (Figure 8). AgNO_3_ (100 μg/ml) showed 63.33 ± 3.05% inhibition and plant extracts were ineffective in inhibiting mycelial growth and spore germination. Changes in the structure of fungal cells could be one of the areas for action of AgNPs. In addition, these particles can destroy macromolecules (DNA and protein), leading to fungal death. Similar studies have reported the antifungal activity of AgNPs against different fungi (Supplementary Table S3) (Khatami et al., 2015; Bahrami-Teimoori et al., 2017; Kumavat and Mishra, 2021).

**TABLE 1:** Mycelial growth inhibition activity of synthesized AgNPs against *F. oxysporum*.

Treatment (µg/mL)	% Inhibition (mycelial growth) *F. oxysporum*
Control	0.00^e^
Plant extract	2.67±1.52^e^
Bavistin	100±0.00^a^
AgNO_3_	63.33±3.05^d^
AgNPs	
50 µg/mL	60.67±2.51^d^
100 µg/mL	72.67±1.52^c^
150 µg/mL	82.00±1.00^b^

Values are means of three independent replicates (*n* = 3).±Indicate standard errors. Means followed by the same letter(s) within the same column are not significantly (*p* ≤ 0.05) different according to Turkey’s HSD.

Jian et al. (2021) suggested that AgNPs can effectively prevent the asexual development of phytopathogenic fungi. Regarding AgNP activity, research on various bacteria and fungi has shown that AgNP treatment can compromise cell membrane integrity and permeability. Furthermore, RNA-Seq data according to the KEGG category showed that AgNPs inhibit the transcription of genes associated with cellular energy expenditure and metabolism in *F. graminearum* (Jian et al., 2021). Similar results were recently observed in another *Fusarium* fungus, suggesting that the disruption of cellular energy expenditure and metabolic pathways is a key component of the antifungal efficacy of AgNPs (Shen et al., 2020).

### Spore germination

The effect of biosynthesized AgNPs on *F. oxysporum* spore germination is shown in Table 2. All concentrations of biosynthesized AgNPs were efficient. A maximum inhibition of spore germination of 73.66 ± 3.94% was observed at a concentration of 150 μg/ml, followed by 100 μg/ml. The optimal concentration of AgNPs in this study was significant compared to the bulk concentration of 100 μg/ml. Similar studies have reported the spore germination activity of AgNPs against different fungi (Sahayaraj et al., 2020; Ahmed et al., 2021; Rizwana and Alwhibi, 2021; Essghaier et al., 2022).

**TABLE 2:** Inhibition activity of AgNPs on spore germination of *F. oxysporum*.

Treatment (%)	% Inhibition (Spore germination) *F. oxysporum*
Control	0.00^c^
AgNO_3_	57.08±6.81^b^
AgNPs	
50 µg/mL	60.77±5.54^b^
100 µg/mL	65.28±2.24^ab^
150 µg/mL	73.66±3.94^a^

Values are means of three independent replicates (*n* = 3). ± indicates standard errors. Means followed by the same letter(s) within the same column do not differ significantly (*p* ≤ 0.05) according to Turkey’s HSD.

## Conclusion

Ensuring a healthy future for nanotechnology requires the application of biogenic synthesis methods for nanoparticle synthesis using o ecologically safe and renewable molecules to avoid the risks associated with the use of hazardous chemical solvents. In this study, we used a simple green approach using *B. aegyptiaca* plant extract to produce stable spherical nanoparticles. The leaf extract functioned as a reducing agent; hence, the synthetic method was superior to traditional methods for preparing AgNPs. The synthesized AgNPs acted as the catalyst to degrade the organic dyes MB and CR with significant efficiency. The poison food approach demonstrated the significant antifungal activity of the synthesized AgNPs against *F. oxysporum.* Using a biosynthetic approach provides new possibilities for the development of the perfect catalyst and antifungal agent with the highest activity and stability.

## Data Availability

The raw data supporting the conclusion of this article will be made available by the authors, without undue reservation.
