# Supplementary material for: Balanites aegyptiaca leaf extract-mediated synthesis of silver nanoparticles and their catalytic dye degradation and antifungal efficacy
Source: Front Bioeng Biotechnol. 2022 Oct 4;10:977101. doi: 10.3389/fbioe.2022.977101 (PMC9576921; doi:10.3389/fbioe.2022.977101)
Supplement: Supplementary file 1 [file Table1.DOCX]

**Supplementary Materials**

Table **S1.** Detail of experiment for catalytic degradation of dyes.

| **Dye** | **Dye’s conc.** | **Volume of dye** | **Volume of H_2_O (DIW)** | **NaBH_4_**  **(0.05M)** | **Volume of AgNPs (0.04%)** | **Reduction time (min)** | **Rate constant (*k*)** |
| --- | --- | --- | --- | --- | --- | --- | --- |
| MB | 1 mM | 50 µL | 2.5 mL | 400 µL | 50 µL | 15 | 0.138 min^-1^ |
| CR | 1 mM | 50 µL | 2.4 mL | 480 µL | 70 µL | 17 | 0.096 min^-1^ |

Table **S2.** Previous study of Dye degradation activity of synthesized AgNPs.

| **S. No.** | **Catalyst** | **Reaction Time (Min.)** | | **References** |
| --- | --- | --- | --- | --- |
|  |  | **CR** | **MB** |  |
| 1 | AgNPs | 18 | 13 | (Bonnia et al., 2016; Varadavenkatesan et al., 2020) |
| 2 | AgNPs | 50 | 20 | (Hamedi et al., 2017; Albeladi et al., 2020) |
| 3 | AgNPs | 9 | 30 | (Sreekanth et al., 2016; Mosaviniya et al., 2019) |
| 4 | AgNPs | 120 | 30 | (Khodadadi et al., 2017; Singh et al., 2019) |
| 5 | AgNPs | 180 | 60 | (Goswami et al., 2018; Althaaly et al., 2022) |
| 6 | AgNPs | 15 | 25 | (Swargiary et al., 2019; Rajkumar et al., 2021) |
| 7 | AgNPs | 9 | 7 | (Naseem et al., 2020; Nouri and Haddioui, 2021) |
| 8 | AgNPs | 17 | 15 | This work |

Table **S3.** Previous study of mycelial growth inhibition activity of synthesized AgNPs.

| **Plants** | **Plant part** | **Zone of inhibition** | **References** |
| --- | --- | --- | --- |
| *Teucrium polium* | leaves | 66.33±2.31mm | (Ghojavand et al., 2020) |
| *Rosa brunonii* | leaves | 65.6% | (Bhagat et al., 2019) |
| *Malva parviflora* | leaves | 54.7% | (Al-Otibi et al., 2021) |
| *Amaranthus retroflexus* | leaves | 53.83 ±1.04 mm | (Bahrami-Teimoori et al., 2017) |
| *Phaseolus vulgaris* | leaves | 58% | (Ege et al., 2020) |
| *Aaronsohnia factorovskyi* | leaves | 85% | (Al-Otibi et al., 2020) |


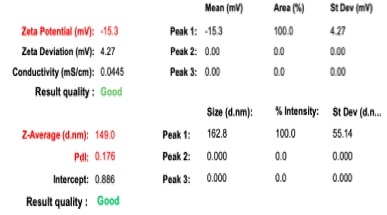


Figure **S2**. (A-C)TEM micrographs (D) d-spacing (E-F) SEM micrographs

Figure **S1.** DLS analysis (Zeta potential and size distribution)

Figure **3S.** Schematic representation of reaction of catalytic reduction of dyes.

**References**

Albeladi, S. S. R., Malik, M. A., and Al-thabaiti, S. A. (2020). Facile biofabrication of silver nanoparticles using Salvia officinalis leaf extract and its catalytic activity towards Congo red dye degradation. *Journal of Materials Research and Technology* 9, 10031–10044.

Al-Otibi, F., Al-Ahaidib, R. A., Alharbi, R. I., Al-Otaibi, R. M., and Albasher, G. (2020). Antimicrobial potential of biosynthesized silver nanoparticles by Aaronsohnia factorovskyi extract. *Molecules* 26, 130.

Al-Otibi, F., Perveen, K., Al-Saif, N. A., Alharbi, R. I., Bokhari, N. A., Albasher, G., et al. (2021). Biosynthesis of silver nanoparticles using Malva parviflora and their antifungal activity. *Saudi J Biol Sci* 28, 2229–2235.

Althaaly, A. F. M., Al-Thabaiti, S. A., and Khan, Z. (2022). Biogenic silver nanoparticles: synthesis, characterization, and degradation of congo red. *Journal of Materials Science: Materials in Electronics*, 1–17.

Bahrami-Teimoori, B., Nikparast, Y., Hojatianfar, M., Akhlaghi, M., Ghorbani, R., and Pourianfar, H. R. (2017). Characterisation and antifungal activity of silver nanoparticles biologically synthesised by Amaranthus retroflexus leaf extract. *J Exp Nanosci* 12, 129–139.

Bhagat, M., Anand, R., Datt, R., Gupta, V., and Arya, S. (2019). Green synthesis of silver nanoparticles using aqueous extract of Rosa brunonii Lindl and their morphological, biological and photocatalytic characterizations. *J Inorg Organomet Polym Mater* 29, 1039–1047.

Bonnia, N. N., Kamaruddin, M. S., Nawawi, M. H., Ratim, S., Azlina, H. N., and Ali, E. S. (2016). Green biosynthesis of silver nanoparticles using ‘Polygonum Hydropiper’and study its catalytic degradation of methylene blue. *Procedia Chem* 19, 594–602.

Ege, E., Kurtay, G., Karaca, B., Büyük, İ., Gökdemir, F. Ş., and Sumer, A. (2020). Green Synthesis of Silver Nanoparticles from Phaseolus vulgaris L. Extracts and Investigation of their Antifungal Activities. *Hacettepe Journal of Biology and Chemistry* 49, 11–23.

Ghojavand, S., Madani, M., and Karimi, J. (2020). Green synthesis, characterization and antifungal activity of silver nanoparticles using stems and flowers of felty germander. *J Inorg Organomet Polym Mater* 30, 2987–2997.

Goswami, M., Baruah, D., and Das, A. M. (2018). Green synthesis of silver nanoparticles supported on cellulose and their catalytic application in the scavenging of organic dyes. *New Journal of Chemistry* 42, 10868–10878.

Hamedi, S., Shojaosadati, S. A., and Mohammadi, A. (2017). Evaluation of the catalytic, antibacterial and anti-biofilm activities of the Convolvulus arvensis extract functionalized silver nanoparticles. *J Photochem Photobiol B* 167, 36–44.

Khodadadi, B., Bordbar, M., and Nasrollahzadeh, M. (2017). Achillea millefolium L. extract mediated green synthesis of waste peach kernel shell supported silver nanoparticles: application of the nanoparticles for catalytic reduction of a variety of dyes in water. *J Colloid Interface Sci* 493, 85–93.

Mosaviniya, M., Kikhavani, T., Tanzifi, M., Yaraki, M. T., Tajbakhsh, P., and Lajevardi, A. (2019). Facile green synthesis of silver nanoparticles using Crocus Haussknechtii Bois bulb extract: Catalytic activity and antibacterial properties. *Colloid Interface Sci Commun* 33, 100211.

Naseem, K., Zia Ur Rehman, M., Ahmad, A., Dubal, D., and AlGarni, T. S. (2020). Plant extract induced biogenic preparation of silver nanoparticles and their potential as catalyst for degradation of toxic dyes. *Coatings* 10, 1235.

Nouri, M., and Haddioui, A. (2021). Improving seed germination and seedling growth of Lepidium sativum with different priming methods under arsenic stress. *Acta Ecologica Sinica* 41, 64–71.

Rajkumar, A., Sivarajasekar, N., and Kandasamy, S. (2021). Bio-Synthesized Silver Nanoparticles For Effective Photo-catalytic Degradation of Congo Red Dye in Aqueous Solutions: Optimization Studies Using Response Surface Methodology. *Analytical Chemistry Letters* 11, 801–815.

Singh, J., Kukkar, P., Sammi, H., Rawat, M., Singh, G., and Kukkar, D. (2019). Enhanced catalytic reduction of 4-nitrophenol and congo red dye By silver nanoparticles prepared from Azadirachta indica leaf extract under direct sunlight exposure. *Particulate Science and Technology* 37, 434–443.

Sreekanth, T. V. M., Jung, M.-J., and Eom, I.-Y. (2016). Green synthesis of silver nanoparticles, decorated on graphene oxide nanosheets and their catalytic activity. *Appl Surf Sci* 361, 102–106.

Swargiary, M., Mitra, A., Halder, D., and Kumar, S. (2019). Fruit extract capped colloidal silver nanoparticles and their application in reduction of methylene blue dye. *Biocatal Biotransformation* 37, 183–189.

Varadavenkatesan, T., Selvaraj, R., and Vinayagam, R. (2020). Green synthesis of silver nanoparticles using Thunbergia grandiflora flower extract and its catalytic action in reduction of Congo red dye. *Mater Today Proc* 23, 39–42.
